# Different pathogenicities of Rice stripe virus from the insect vector and from viruliferous plants

**DOI:** 10.1111/nph.13747

**Published:** 2015-11-20

**Authors:** Wan Zhao, Pengcheng Yang, Le Kang, Feng Cui

**Affiliations:** ^1^ State Key Laboratory of Integrated Management of Pest Insects and RodentsInstitute of ZoologyChinese Academy of SciencesBeijingChina; ^2^ Beijing Institutes of Life ScienceChinese Academy of SciencesBeijingChina

**Keywords:** alimentary canal, chloroplast, mechanical inoculation, rice stripe disease, salivary gland, small brown planthopper (*Laodelphax striatellus*)

## Abstract

- Persistent plant viruses usually depend on insects for their transmission; they cannot be transmitted between plants or through mechanical inoculation. However, the mechanism by which persistent viruses become pathogenic in insect vectors remains unknown. In this study, we used Rice stripe virus (RSV), its insect vector *Laodelphax striatellus* and host plant (*Oryza sativa*) to explore how persistent viruses acquire pathogenicity from insect vectors.
- RSV acquired phytopathogenicity in both the alimentary tract and the salivary gland of *L. striatellus*.
- We mechanically inoculated RSV into rice *O. sativa* leaves through midrib microinjection. Insect‐derived RSV induced a typical stripe symptom, whereas plant‐derived RSV only produced chlorosis in rice leaves. Insect‐derived RSV had higher expression of genes *rdrp*,* ns2*,* nsvc2*,* sp* and *nsvc4* than plant‐derived RSV, and the latter had higher expression of genes *cp* and *ns3* than the former in rice leaves. Different from plant‐derived RSV, insect‐derived RSV damaged grana stacks within the chloroplast and inhibited photosynthesis by suppressing the photosystem II subunit *psbp*.
- This study not only presented a convenient method to mechanically inoculate RSV into plants, but also provided insights into the different pathogenic mechanisms of RSV from the insect vector and from viruliferous plants.

## Introduction

Approximately 80% of plant viruses depend on insect vectors for transmission (Andret‐Link & Fuchs, 2005; Thomas, 2007). More than 200 plant viruses are transmitted through hemipteran insects, such as aphids, whiteflies, leafhoppers, planthoppers and thrips (Hogenhout *et al*., 2008). Viruses can be grouped into three categories on the basis of their transmission mode: nonpersistent, semipersistent and persistent (Nault & Ammar, 1989). Insects transmit persistent viruses throughout their lifespan. Persistent viruses transfer from the alimentary canals to the salivary glands of vectors and then become ejected upon feeding (Ammar, 1994; Ammar & Hogenhout, 2008). Persistent viruses can be classified as persistent‐circulative (mostly nonpropagative) or persistent‐propagative on the basis of their replication features (Nault, 1997). Most persistent‐propagative viruses (e.g. *Rhabdoviruses*,* Reoviruses*,* Marafiviruses* and *Tenuiviruses*) cannot be transmitted to plants by seeds or by introducing biologically active plant‐derived viruses into living cells through wounds or abrasions on the plant surface (Louie, 1995; Madriz‐Ordenana *et al*., 2000). This finding indicates that viruses are extremely dependent on and share complicated interactions with insect vectors. However, the mechanism by which persistent viruses become pathogenic in insect vectors remains unknown.

Rice stripe virus (RSV), a single‐stranded RNA virus of the genus *Tenuivirus*, causes rice stripe disease, which is one of the most notorious rice diseases in temperate and subtropical regions (Toriyama, 1986; Falk & Tsai, 1998). RSV uses a negative‐sense and ambisense coding strategies to encode seven proteins, that is, RNA‐dependent RNA polymerase (RdRp), NS2, NSvc2 (putative membrane glycoprotein), NS3 (gene silencing suppressor), Cp (nucleocapsid protein), Sp (nonstructural disease‐specific protein) and NSvc4 (movement protein) (Kakutani *et al*., 1990, 1991; Zhu *et al*., 1992; Takahashi *et al*., 1993; Toriyama *et al*., 1994; Xiong *et al*., 2008, 2009). RSV is efficiently transmitted by the small brown planthopper *Laodelphax striatellus* in a persistent‐propagative manner (Toriyama, 1986). Typical symptoms of RSV include pale and discontinuous yellow stripes, blotches and dead tissue streaks on the leaves.

In general, RSV extracted from viruliferous rice cannot infect healthy rice through mechanical inoculation despite the various inoculation buffers, conditions, techniques and inoculum sources (young leaves and rootlets from field‐ and laboratory‐infected plants) that have been utilized (Ling, 1972). However, whether or not the nonpathogenicity of plant‐derived RSV is caused by the improper inoculation methods or by the lack of required modifications in insect vectors remains unknown.

The different pathogenicities of RSV from insect vectors and viruliferous plants might be caused by the modifications of RSV in insect vectors. Persistent‐propagative viruses first accumulate in the alimentary canal, transfer into the hemolymph or nervous tissues across the epithelial cell walls, propagate in specific organs and then arrive at the salivary gland, from which viruses are inoculated back to the plant hosts during feeding (Ammar, 1994; Ammar & Hogenhout, 2008). Before transmission, persistent viruses require a latent period (the incubation period), which is the time between the acquisition and inoculation access periods. The latent period ranges from hours to weeks (Ammar & Hogenhout, 2008). RSV accumulates in the epithelial cells of the midgut, fat bodies and follicular cells of the ovariole, ovary and principal salivary gland (Suzuki *et al*., 1992; Liang *et al*., 2005; Huo *et al*., 2014). The alimentary canal and salivary glands are potential virus replication sites. The latent period of RSV in small brown planthoppers ranges from 3 to 10 d (Hsieh, 1974). However, whether or not the pathogenicity acquirement is involved in specific organs is unknown. The molecular reactions induced by RSV in the different organs of insects are also unclear, although the transcriptional profiles of viruliferous and nonviruliferous small brown planthoppers have been compared in the whole insect body (Zhang *et al*., 2010).

In order to explore the nature of acquiring phytopathogenicity by persistent viruses in insect vectors, in this study, insect‐derived RSV is compared with plant‐derived RSV in disease symptom development, virus activity during infection, molecular response by plants and effect on chloroplast structure. Virus access, propagation, latent period and acquiring pathogenicity in the alimentary canal and the salivary gland, as well as the molecular response of the two organs to RSV, are also clarified.

## Materials and Methods

### Insect rearing

The viruliferous and nonviruliferous small brown planthopper (*Laodelphax striatellusi*, Fallen) strains used in this study were collected from a field population in Hai'an, Jiangsu Province, China and maintained in the laboratory for nearly 7 yr. The planthoppers were reared on 2–3 cm seedlings of rice *Oryza sativa* L. spp. *japonica* var. *nippobare* in glass incubators, which were sealed with a nylon mesh at 25°C, with 16 h of light daily. The insects were transferred to fresh rice seedlings every 8 d to ensure sufficient nutrition. The presence of RSV was discerned via dot‐ELISA with the monoclonal anti‐Cp antibody (Wang *et al*., 2004). The RSV‐carrying rate of the viruliferous strain was maintained at ≥ 90% through a purification selection every 3 months with dot‐ELISA.

### RNA isolation and cDNA synthesis

RNA was isolated from rice plants or small brown planthoppers by using TRIzol Reagent (Invitrogen) in accordance with the handbook instruction. Approximately 100 mg of rice leaves or 30 organs (salivary gland or alimentary canal) of small brown planthoppers were ground in liquid nitrogen and mixed with 1 ml of TRIzol Reagent for RNA extraction. The concentration and quality of RNA were measured using a NanoDrop spectrophotometer (Thermo Scientific, Waltham, MA, USA) and through gel electrophoresis. RNA was treated using the TURBO DNA‐free kit (Ambion) to remove genomic DNA contamination before using in cDNA synthesis. RNA (1 μg) was reverse‐transcribed to cDNA using the Superscript III First‐Strand Synthesis System (Invitrogen) and random primers (Promega) in accordance with the manufacturer's instructions.

### Isolation and quantification of RSV crude preparations

RSV‐infected rice seedlings (100 mg), 30 whole bodies, 100 alimentary canals or 500 salivary glands of viruliferous small brown planthoppers were ground in liquid nitrogen and then mixed with PBS‐EDTA buffer (containing 0.15 M NaCl, 0.01 M PBS solution, 0.01 M EDTA and 0.5% 2‐mercaptoethanol, pH 7.2). After centrifugation at 1344 ***g*** for 5 min and at 8400 ***g*** for 15 min at 4°C, the supernatant containing RSV was collected and stored at 4°C for further usage. Crude extracts from healthy rice seedlings and nonviruliferous small brown planthoppers were isolated using the same protocol as negative controls. The presence of RSV in the crude preparations was verified via dot‐ELISA with the monoclonal anti‐Cp antibody (Wang *et al*., 2004). The concentration of RSV in crude preparations was measured using ELISA (Howie & Thorsen, 1981) and Western bolt. The crude extracts from healthy planthoppers or healthy rice were used as the negative controls, respectively, in order to avoid the interference from different host environment. The anti‐mouse IgG, HRP‐conjugated antibody (1 : 5000) and substrate chromogen TMB mixture (1 mg of TMB, and 10 μl of H_2_O_2_ in 9.9 ml of phosphoric acid‐citric acid buffer pH 5.0) were sequentially added. The OD_450_ values were recorded on SpectraMax 340PC384 Microplate Reader (Molecular Devices, Sunnyvale, CA, USA).

### Feeding small brown planthoppers with RSV crude preparations

RSV crude preparations (20 μl) from viruliferous small brown planthoppers or RSV‐infected rice seedlings were mixed with 100 μl of artificial diet (Fu *et al*., 2001). The artificial diet containing the PBS‐EDTA buffer was used as a negative control. Nonviruliferous fifth‐instar nymphs were transferred into a sterile plastic cap (Falcon, Primaria, NJ, USA), which was then covered with a layer of parafilm. Subsequently, 120 μl of artificial diet containing RSV crude preparations was added on top of the parafilm, and a second layer of parafilm was placed to form a feeding sachet. The planthoppers were fed for a particular period and then transferred to healthy rice seedlings.

### Determination of the minimum acquisition access period of RSV

Nonviruliferous fifth‐instar nymphs were fed on the artificial diet containing plant‐derived RSV crude preparations for 1, 3 or 5 min. The alimentary canals and primary salivary glands of the nymphs were dissected in 0.9% RNA‐free saline solution for RNA extraction and cDNA synthesis. The RNA level of the RSV *cp* gene in each tissue was measured via quantitative reverse transcription PCR (qRT‐PCR). Four replicates and 30 tissues per replicate were prepared. RSV was visualized in the two organs at 3 or 5 min access period through immunohistochemistry. The tissues from six individuals were fixed in 4% paraformaldehyde in PBS for 2 h at room temperature and then incubated in osmotic buffer (2% triton in PBS) for 4 h. The Alexa Fluor 488‐labeled anti‐CP monoclonal antibody was added for 2 h incubation. The nucleus was labeled with Hoechst in accordance with the manufacturer's instruction (Invitrogen). The images were viewed under a Leica TCS SP5 confocal microscope (Leica Microsystems, Solms, Germany).

### Quantification of RSV proliferation in the alimentary canals and salivary glands of small brown planthoppers

Nonviruliferous fifth‐instar nymphs were fed on the artificial diet containing plant‐derived RSV crude preparations for 3 min and then raised on healthy rice seedlings. Thirty insects were collected at 15 min, 30 min, 1 h, 3 h, 6 h, 12 h, 24 h and then every 24 h until 7 d for alimentary canal and salivary gland dissection. Four biological replicates were prepared. Total RNAs were isolated from the two tissues at each time point, and the relative RNA level of *cp* was determined by qRT‐PCR. RSV crude extracts from the two tissues at 12, 48, 96, 120 and 144 h were isolated for RSV content measurement by Western blot analysis using monoclonal anti‐Cp antibody. Beta‐tubulin of small brown planthoppers was used as an internal control by immunoblotting against the monoclonal anti‐beta‐tubulin antibody (CW0098; CWBIO, Beijing, China). The immune signal was visualized using Image Station 4000MM ProCFL (Carestream, Rochester, NY, USA), and the signal gray was analyzed with the ImageJ online server (http://rsb.info.nih.gov/ij/). The RSV content was quantified as the gray ratio between Cp and beta‐tubulin.

### Determination of RSV latent period in small brown planthoppers

After feeding on the artificial diet containing plant‐derived RSV crude preparations for 3 min, five virus‐acquired fifth‐instar nymphs each were raised on healthy rice seedlings for 12, 48, 96, 120 or 144 h, and then transferred to new healthy rice seedlings for 15 min of virus inoculation. In total, 10 plants were tested. After removal of small brown planthoppers, the inoculated seedlings were cultured at 25°C for disease symptom observation within 6–8 wk. The RNA and protein levels of the RSV *cp* gene in rice seedlings were measured via reverse transcription PCR (RT‐PCR) and Western blot analysis after 3 wk of culture. RT‐PCR was run on the Mastercycler thermal cycler (Eppendorf, Hamburg, Germany) under cycling conditions of 95°C for 5 min, followed by 30 cycles of 95°C for 20 s, 55°C for 30 s and 72°C for 30 s. Primer pairs *cp*‐F/*cp*‐R and *ubq*‐q‐F/*ubq*‐q‐R were used to amplify RSV *cp* (DQ299151) and rice *ubiquitin* (AK061988) as internal controls (Supporting Information Table S1), respectively. PCR products were separated on 1% agarose gel and viewed with Syngene G: BOX gel imaging and analysis system (Syngene, Cambridge, UK) after being stained with GelRed (Biotium, Hayward, CA, USA). Cp was detected by Western blot analysis using monoclonal anti‐Cp antibody. Beta‐actin of rice was used as an internal control by immunoblotting against plant β‐actin monoclonal antibody (KM9004; Sungene Biotech, Tianjin, China).

### Mechanical inoculation of RSV in rice seedlings

RSV crude preparations that contained comparable amounts of RSV from viruliferous small brown planthoppers, the salivary glands and alimentary canals of planthoppers infected with RSV for 96 h or 120 h, or RSV‐infected rice seedlings were microinjected into the midribs of healthy 2‐wk‐old rice leaves through a glass needle at slow speed using Nanoliter 2000 (World Precision Instruments, Sarasota, FL, USA). The concentration of RSV in crude preparations was verified by Western blot analysis using monoclonal anti‐Cp antibody. Beta‐actins of rice and planthoppers were used as internal controls by immunoblotting against the β‐actin monoclonal antibody (MA5‐15739; Pierce, Rockford, IL, USA). Each leaf was microinjected five times with 23 nl of preparations for each time in an interval of 1 cm on the midrib. Five dilution proportions (from 1 : 1 to 1 : 10 000) of RSV crude preparations from RSV‐infected rice seedlings were also injected. Extracts from nonviruliferous small brown planthoppers or healthy rice leaves were injected into the midribs of healthy rice leaves as negative controls. Each group contained 30 seedlings, and three replicates were prepared. Both the injected leaves and systemic leaves were evaluated for the development of disease symptoms and the presence of RSV using the monoclonal anti‐Cp antibody in Western blot analysis. Beta‐actin of rice was used as an internal control by immunoblotting against plant β‐actin monoclonal antibody (KM9004; Sungene Biotech).

### Colloidal gold immunoelectron microscopy

Leaves of healthy rice, leaves fed on by viruliferous small brown planthoppers or leaves mechanically inoculated with insect‐derived RSV or plant‐derived RSV were fixed in 50 mM PBS buffer containing 1% glutaraldehyde and 2% formaldehyde (pH 6.8) at 4°C for 3 h. After dehydration in 30%, 50%, 70%, 90%, 95% and 100% alcohol, the leaves were embedded in LR Gold Resin (Fluka Biochemika, Steinheim, Switzerland). Ultrathin sections of the embedded leaves were cut and placed onto 100‐mesh nickel grids and then blocked for 30 min in 50 mM PBS buffer containing 3% goat serum and 0.02% polyethylene glycol 2000. The anti‐Cp monoclonal antibody (1 : 1000) and 10‐nm gold‐conjugated goat‐anti‐mouse IgG (1 : 100; Sigma‐Aldrich) were sequentially added for 2 h incubation. The grids were stained in 2% neutral uranyl acetate for 20 min. The sections were viewed under a JEM‐1400 transmission electron microscope (JEOL, Tokyo, Japan) at 80 kV accelerating voltage. Six leaves and three sections of each leaf for each group were observed.

### Total soluble sugar content measurement

Total soluble sugar content was measured based on the anthrone colorimetric method (Irigoyen *et al*., 1992). One gram (g) of the nonviruliferous rice leaves, leaves infected by viruliferous planthoppers, or leaves mechanically inoculated with insect‐ or plant‐derived RSV were crushed in a mortar with liquid nitrogen and 5 ml of 80% alcohol was added. The mixture was heated in an 80°C metal bath for 30 min, and then centrifuged at 3024 ***g*** for 5 min. The supernatant was collected and 100 μl of the extraction was mixed with 500 μl of anthrone reagent. The mixture was heated in a metal bath at 100°C for 10 min, then the reaction was terminated on ice for 5 min. The absorbance at 620 nm wavelength (OD_620_) was measured. The total soluble sugar content was calculated based on a glucose standard curve. Four replicates were prepared. The content was reported as mean ± SE. Differences were statistically evaluated using SPSS 17.0 (SPSS Inc., Chicago, IL, USA). One‐way ANOVA followed by a Tukey's test was applied for multiple comparisons.

### Transcriptome sequencing and analysis

The rice leaves of healthy plants, those fed on by viruliferous small brown planthoppers, or leaves mechanically inoculated with insect‐derived RSV, plant‐derived RSV, extracts of nonviruliferous small brown planthoppers or extracts of healthy plants were collected after 3 wk of inoculation for single‐end digital gene expression sequencing using Illumina short‐read sequencing. Three biological replicates and five leaves per replicate were prepared for each group. At least 10 million clean reads were produced for each rice sample. Reads of each sample were deposited in the Short Read Archive of the National Center for Biotechnology Information (NCBI) with accession numbers of SRP049001 (*O. sativa*).

The RNA‐seq reads of rice samples were mapped using Tophat2 (v.2.0.10) (Kim *et al*., 2013). The number of reads mapped to every gene model was counted using Htseq (Anders *et al*., 2015). The gene annotation files, including gene structure gff, CDS and protein sequence files, were downloaded from ftp://ftp.plantbiology.msu.edu/pub/data/Eukaryotic_Projects/o_sativa/annotation_dbs/pseudomolecules/version_7.0/. The multiple isoforms belonging to one gene were filtered, with the longest isoform as representative. The edgeR package was used to detect the differentially expressed genes with a fold change cutoff of at least two and FDR cutoff value of 0.01. Expression‐based sample clustering was performed using the heatmap.2 function implemented in R package gplots (v.2.16.0) (Warnes *et al*., 2009). Before clustering, genes with reads per kilobase per million reads (RPKM) of less than five at any sample were filtered, and the RPKM values were log_2_‐transformed and normalized to mean zero and variance one.

Kyoto Encyclopedia of Genes and Genomes (KEGG) enrichment analysis for the differentially expressed genes was performed, with the whole annotated gene set as the background. The *P*‐value was approximated using the Chi‐square test. Fisher's exact test was applied when any expected value of count was below five. To adjust for multiple testing, FDR was calculated for each class using the Benjamini–Hochberg method (Benjamini & Hochberg, 1995).

### Quantitative real‐time PCR

Quantitative real‐time (qRT)‐PCR was used to quantify the relative RNA levels of seven RSV genes (*rdrp*, JQ927433; *ns2*, EF493228; *nscv2*, NC_003754; *ns3*, EF493242; *cp*, DQ299151; *sp*, AJ578465; and *nsvc4*, ABC68339) and the transcript level of rice *psbp* gene (KF460579) in the rice leaves mechanically inoculated with a comparative amount of insect‐derived RSV or plant‐derived RSV. The relative RNA level of *cp* in the salivary glands and alimentary canals of small brown planthoppers and the *PsbP* transcript in the rice leaves of healthy plants, those fed on by viruliferous small brown planthoppers, or those mechanically inoculated with extracts of nonviruliferous planthoppers or extracts of healthy plants were also quantified. Table S1 lists the primers used to amplify each gene, and the PCR products were confirmed by sequencing. qRT‐PCR was carried out in 20 μl of reaction agent composed of 2.5 μl of template cDNA, 10 μl of 2 × SYBR Green PCR Master Mix (Fermentas, Waltham, MA, USA, and 0.25 μM each primer on Light Cycler 480 II (Roche). The thermal cycling conditions were as follows: 95°C for 2 min, followed by 40 cycles of 95°C for 30 s, 60°C for 30 s and 68°C for 40 s. The transcript levels of small brown planthopper translation elongation factor 2 (*ef2*) (Zhang *et al*., 2012) and rice *ubiquitin* (AK061988) were quantified with primer pairs *ef2*‐q‐F/*ef2*‐q‐R and *ubq*‐q‐F/*ubq*‐q‐R to normalize the cDNA templates of planthoppers and rice leaves, respectively (Table S1). The relative transcript level of each gene was reported as mean ± SE. Differences were statistically evaluated using SPSS 17.0. Student's *t*‐test was performed to compare two means, whereas one‐way ANOVA followed by a Tukey's test was applied for multiple comparisons.

## Results

### Acquisition, inoculation and pathogenicity of RSV in the alimentary canal and salivary gland of small brown planthoppers

**Figure 1:**
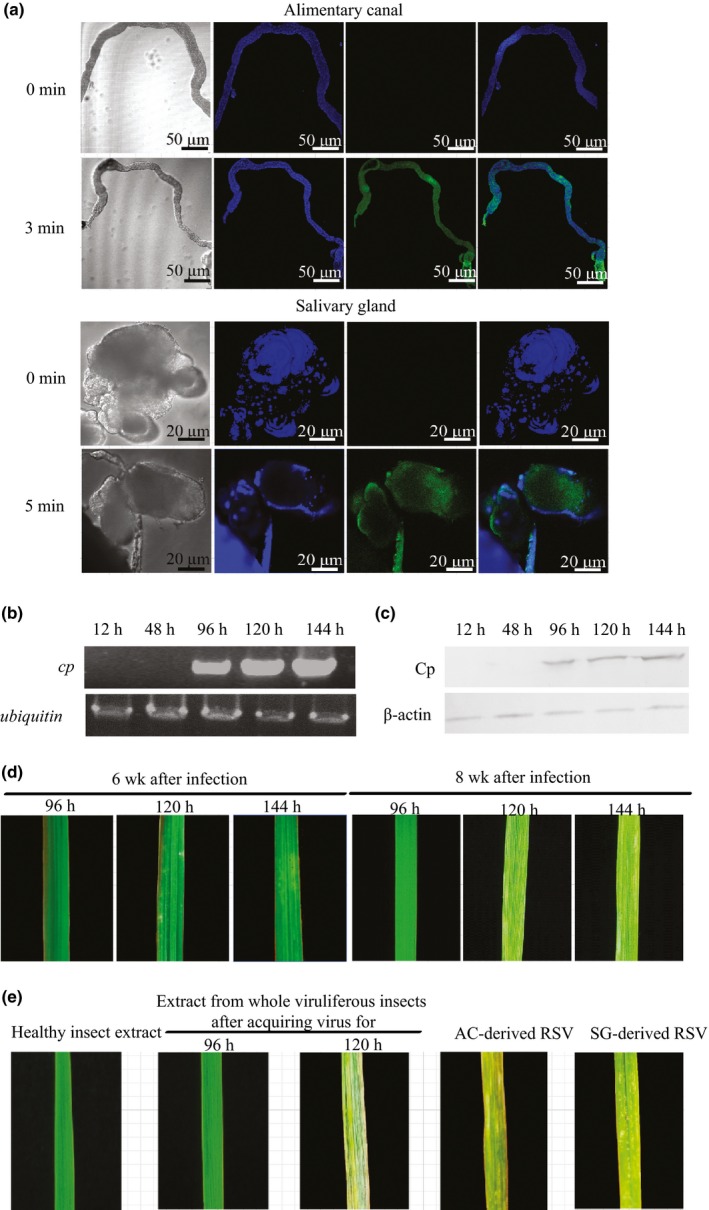
Acquisition, inoculation and pathogenicity of Rice stripe virus (RSV) in the alimentary canal and salivary gland of small brown planthoppers (*Laodelphax striatellus*). (a) Minimum acquisition access period of RSV in the alimentary canal and salivary gland visualized through immunohistochemistry at 3 or 5 min after small brown planthoppers were fed on the artificial diet containing plant‐derived RSV crude preparations. Green signal is from Alexa Fluor 488‐labeled anti‐Cp monoclonal antibody, and blue signal is the nucleus stained with Hoechst. (b, c) The RNA levels of *cp* gene and protein levels of Cp in rice leaves (*Oryza sativa*) inoculated by small brown planthoppers that acquired RSV for 12, 48, 96, 120 or 144 h, viewed via qRT‐PCR and Western blot analysis, respectively. Rice *ubiquitin* (AK061988) and plant beta‐actin were used as internal controls. (d) Disease symptoms of rice leaves inoculated by small brown planthoppers that acquired RSV for 96, 120 or 144 h after 6 and 8 wk. (e) Disease symptom of rice leaves microinjected with RSV crude preparations from the whole viruliferous planthoppers after acquiring the virus for 96 h or 120 h, or from the salivary glands (SG) or alimentary canals (AC) of the insects after acquiring virus for 120 h. Healthy insect crude extracts served as negative control.

RSV was first detected in the alimentary canal 3 min after fifth‐instar naive nymphs were fed on artificial diets that contain crude RSV preparations. Thus, the minimum acquisition access period of RSV was 3 min in small brown planthoppers. RSV was also detected in the salivary glands after 5 min (Figs 1a, S1a). When the RSV‐borne nymphs were transferred to and raised on healthy rice seedlings, the *cp* RNA level showed two peaks at 48 and 120 h in the alimentary canal and one at 1 wk in the salivary gland (Fig. S1b). The protein level of Cp peaked at 120 h in both organs, but the amount of Cp in the alimentary canal was higher than that in the salivary gland (Fig. S1c,d). RSV transferred to healthy rice seedlings after 96 h (Fig. 1b,c), indicating that the latent period of RSV in small brown planthoppers was 96 h. However, RSV only caused the typical stripe symptom in rice leaves after 120 h or longer development in small brown planthoppers, regardless of whether it was transmitted by planthoppers or by microinjection (Fig. 1d,e). RSV from the alimentary canal and the salivary gland were tested by the mechanical inoculation of 120 h‐developed viruses. Both the alimentary canal‐derived and the salivary gland‐derived RSVs led to the typical symptom of rice stripe disease, suggesting the non‐tissue‐specific pathogenicity of RSV from the insects (Fig. 1e).

### Different pathogenicities of RSV from insect vectors and viruliferous rice

**Figure 2:**
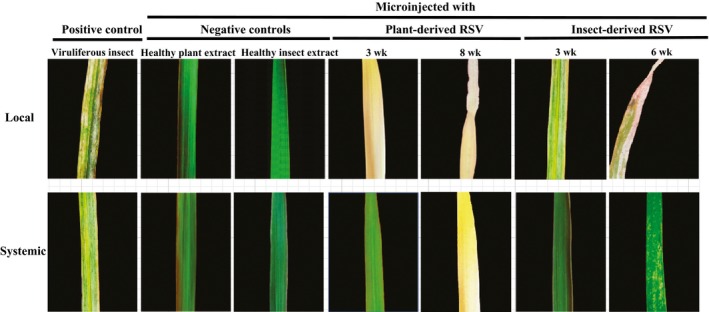
Disease symptoms of rice leaves (*Oryza sativa*) inoculated by viruliferous small brown planthoppers (*Laodelphax striatellus*) or microinjected with Rice stripe virus (RSV) crude preparations from RSV‐infected rice seedlings or viruliferous small brown planthoppers. Crude extracts from nonviruliferous small brown planthoppers or healthy rice leaves served as negative controls.

Crude RSV preparations were isolated from infected planthoppers and rice leaves. After quantifying the protein Cp level using ELISA and Western blot (Fig. S2a) in the RSV preparations, comparable amounts of RSV from the two sources were microinjected into healthy rice leaves. As controls, crude extracts from healthy plants or nonviruliferous small brown planthoppers were also microinjected. After 3 wk, *c*. 17% (± 3%) of the rice seedlings inoculated with insect‐derived RSV extracts displayed typical discontinuous yellow stripes in the injected leaves. The stripe symptom systemically appeared after 6 wk (Fig. 2). The rate of RSV infection by mechanical inoculation was lower than that by viruliferous planthopper transmission, which was *c*. 53% (± 13%). When rice‐derived RSV was mechanically inoculated, no typical stripe symptom was observed, but 23% (± 10%) of the rice seedlings turned pale yellow‐green in the whole injected leaves after 3 wk and systemically after 8 wk (Fig. 2). The extent of chlorosis was RSV dosage‐dependent (Fig. S2c), but no typical stripe symptom was observed at any amount of virus load. The rice‐derived RSV extract, whether in the artificial diet or injected in rice, induced the typical stripe symptom only after being fed on and transmitted by planthoppers (Fig. S2d,e). No chlorosis or stripe symptom was observed in the rice seedlings that were mechanically inoculated or insect‐inoculated with the crude extracts from healthy plants or nonviruliferous planthoppers (Figs. 2, S2d) Therefore, insect‐derived RSV can cause typical rice stripe symptoms by mechanical inoculation, whereas plant‐derived RSV without insect vectors can only induce rice chlorosis.

### Activity of insect‐derived and plant‐derived RSV in rice

**Figure 3:**
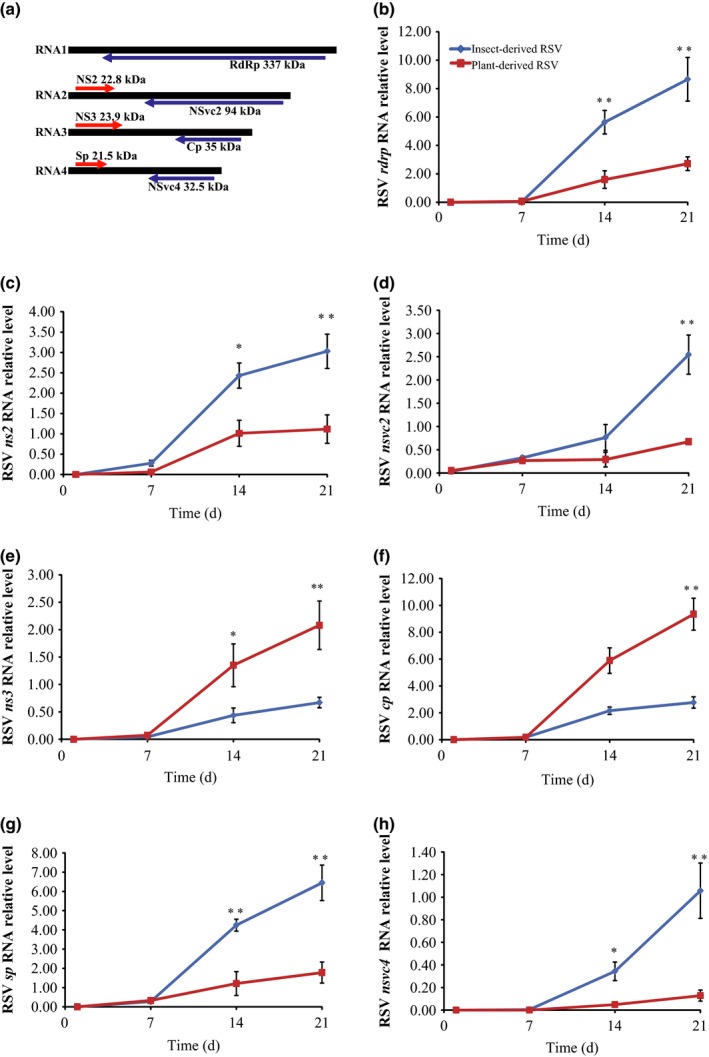
Activity of insect‐ and plant‐derived Rice stripe virus (RSV) in rice leaves (*Oryza sativa*) inoculated through microinjection. (a) Genome structure and seven coding regions of RSV. Arrows indicate the position and transcription direction of each coding gene. (b–h) The RNA levels of the seven genes of RSV were measured using qRT‐PCR as a function of time. Rice *ubiquitin* (AK061988) was quantified to normalize the cDNA templates of rice leaves. The RNA level of each gene was reported as mean ± SE. Differences were statistically evaluated by Student's *t*‐test using SPSS 17.0. *, *P *<* *0.05; **, *P *<* *0.01.

The activities of insect‐ and plant‐derived RSV mechanically inoculated in rice leaves were compared by measuring the RNA levels (containing mRNA and genomic RNA) of seven RSV genes until 3 wk after inoculation (Fig. 3b–h). In the first week, the two origins of RSV showed similar RNA levels of the seven genes, indicating a comparable level of viral inoculation. After 1 wk, we observed that the RNA level of *cp* from the two origins of RSV increased. Insect‐derived RSV had higher RNA levels of the genes *rdrp*,* ns2*,* nsvc2*,* sp* and *nsvc4* than plant‐derived RSV, whereas the latter had higher expression levels of the genes *cp* and *ns3*. Based on the genome location of the seven viral genes, we can conclude that insect‐derived RSV showed a higher expression activity in the genome segments of RNA1, RNA2 and RNA4 than plant‐derived RSV during plant infection. It should be noted that some of the differences in expression between plant‐ and insect‐derived RSV may be due to differences in replication of the viral genomes (RNA1–RNA4).

### Different molecular responses of rice leaves to the infection of insect‐ and plant‐derived RSV

**Figure 4:**
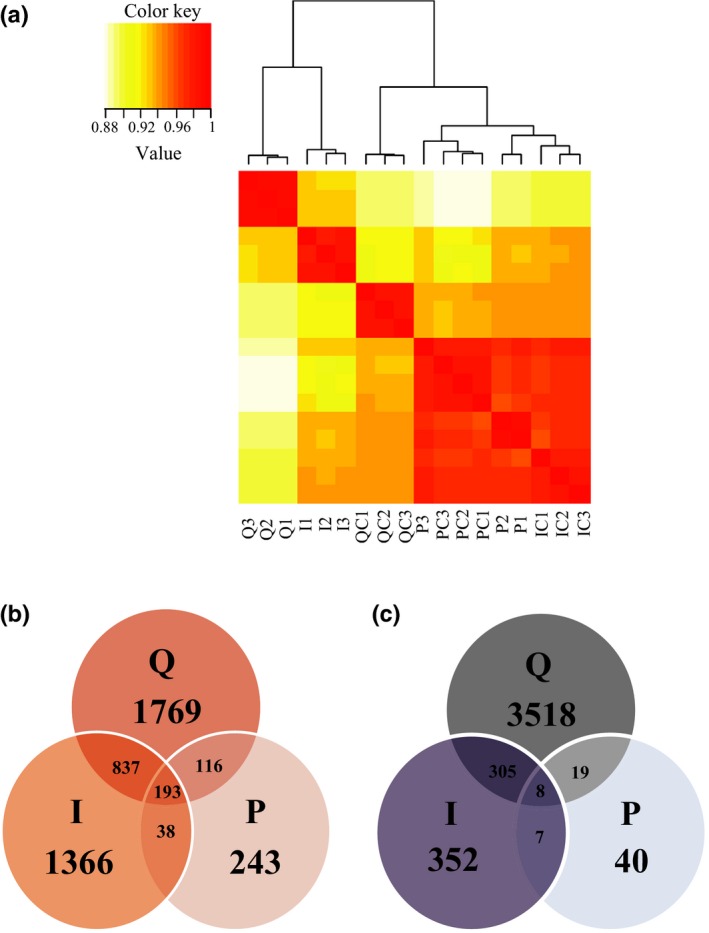
Transcriptome comparison among the rice leaves (*Oryza sativa*) inoculated by viruliferous small brown planthoppers (*Laodelphax striatellus*) or microinjected with Rice stripe virus (RSV) crude preparations from RSV‐infected rice seedlings or viruliferous small brown planthoppers. (a) Pearson correlation analysis of transcriptional profiles using the heatmap.2 function implemented in R package gplots. (b, c) Venn diagrams illustrating the number of upregulated (b) and downregulated genes (c) in the three treatments of rice leaves compared with their respective controls. Three biological replicates were sequenced for each group. Q, rice leaves fed on by viruliferous planthoppers; QC, rice leaves without planthopper infestation; I, rice leaves microinjected with insect‐derived RSV; IC, rice leaves microinjected with extracts of nonviruliferous planthoppers; P, rice leaves microinjected with plant‐derived RSV; PC, rice leaves microinjected with extracts of healthy plants.

Large‐scale gene expression analysis was conducted on rice leaves that were mechanically inoculated with insect‐ or plant‐derived RSV, or were fed on by viruliferous planthoppers. The corresponding controls were rice leaves that were mechanically inoculated with extracts of healthy planthoppers or rice leaves, or rice leaves without infection of planthoppers. At least 10 million clean reads were obtained for each sample, and the Q30 value was > 97% (Table S2). At the whole transcriptome level, the gene expression pattern of RSV‐infected leaves by small brown planthoppers clustered with that of leaves mechanically inoculated with insect‐derived RSV; furthermore, the two treatments were outgrouped from the treatment of plant‐derived‐RSV inoculation and various controls (Fig. 4a). Compared with their respective controls, 6765, 3106 and 583 genes were differentially expressed in the leaves fed on by viruliferous planthoppers or mechanically inoculated with insect‐ or plant‐derived RSV, respectively. The number of co‐regulated genes between the leaves fed on by viruliferous planthoppers and mechanically inoculated with insect‐derived RSV was the most among the three pairwise comparisons (Fig. 4b,c).

**Figure 5:**
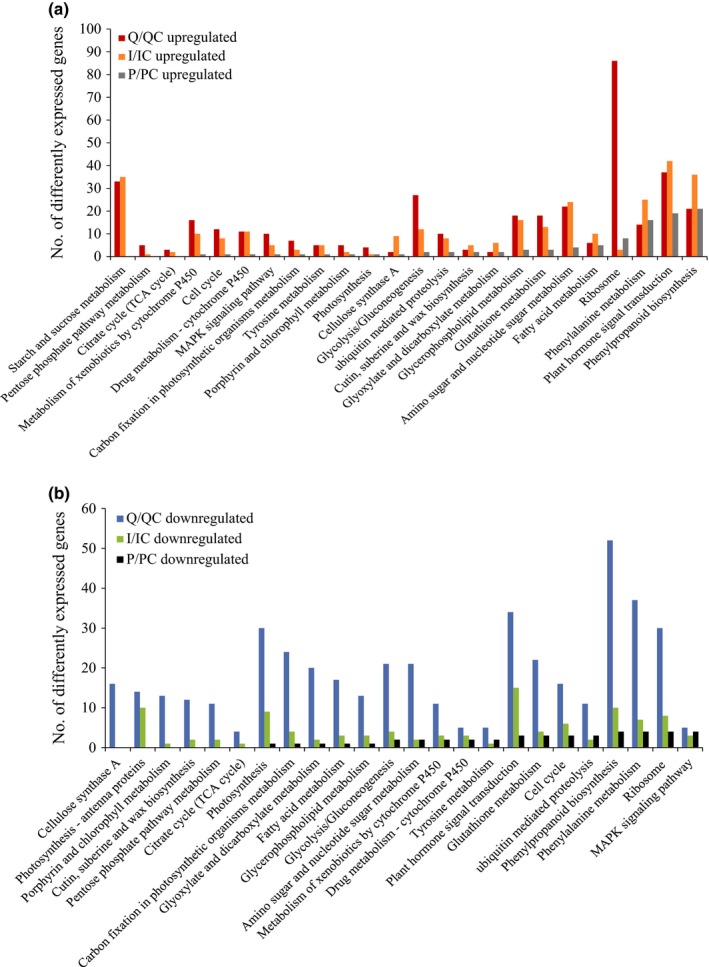
Kyoto Encyclopedia of Genes and Genomes (KEGG) analysis of (a) upregulated and (b) downregulated genes in rice leaves (*Oryza sativa*) after Rice stripe virus (RSV) infection. Q, rice leaves fed on by viruliferous planthoppers (*Laodelphax striatellus*); QC, rice leaves without planthopper infestation; I, rice leaves microinjected with insect‐derived RSV; IC, rice leaves microinjected with extracts of nonviruliferous planthoppers; P, rice leaves microinjected with plant‐derived RSV; PC, rice leaves microinjected with extracts of healthy plants.

**Figure 6:**
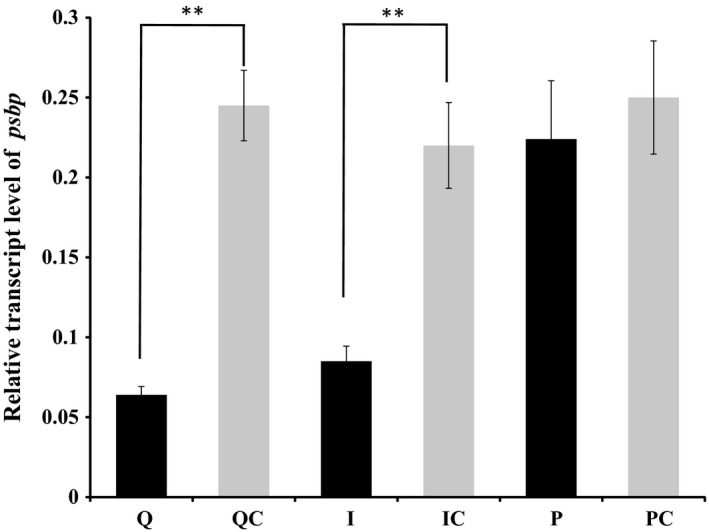
Relative transcript level of the *psbp* gene in rice leaves (*Oryza sativa*) quantified with quantitative real‐time PCR. Rice *ubiquitin* (AK061988) was quantified to normalize the cDNA templates of rice leaves. The relative transcript level of *psbp* was reported as mean ± SE. Differences were statistically evaluated by Student's *t*‐test using SPSS 17.0. **, *P *<* *0.01. Q, rice leaves fed on by viruliferous planthoppers (*Laodelphax striatellus*); QC, rice leaves without planthopper infestation; I, rice leaves microinjected with insect‐derived RSV; IC, rice leaves microinjected with extracts of nonviruliferous planthoppers; P, rice leaves microinjected with plant‐derived RSV; PC, rice leaves microinjected with extracts of healthy plants.

Among the differently expressed genes, 77 involved in starch and sucrose metabolism, glycolysis/gluconeogenesis, amino sugar and nucleotide sugar metabolism were specifically upregulated in the leaves fed on by viruliferous planthoppers or mechanically inoculated with insect‐derived RSV (Fig. 5a). Meanwhile, 26 genes participating in photosynthesis and in porphyrin and chlorophyll metabolism were particularly downregulated in the leaves fed on by viruliferous planthoppers or mechanically inoculated with insect‐derived RSV, but not in the leaves inoculated with plant‐derived RSV (Fig. 5b). These downregulated photosynthesis‐related genes encode light‐harvesting complex I Chl*a*/*b* binding protein 1–4, light‐harvesting complex II Chl*a*/*b* binding protein 1–6, photosystem II subunits (PsbO, PsbP, PsbR and PsbY), photosystem I subunits (PsaG, PsaK, PsaN and PsaO), ATPase subunit b and ferredoxin (Table S3). The photosystem II subunit PsbP, which is a component of the luminal protein complex (Sato, 2010), is important for maintaining the integrity of grana stacks in chloroplasts (Yi *et al*., 2007; Roose *et al*., 2011). Decreasing *psbp* expression in plants aggravates disease symptom (Kong *et al*., 2014). The qRT‐PCR demonstrated that *psbp* transcription decreased by 76% and 61% in the leaves fed on by viruliferous planthoppers and the leaves mechanically inoculated with insect‐derived RSV, respectively, but not in the group mechanically inoculated with plant‐derived RSV (Fig. 6).

### Different effects of insect‐ and plant‐derived RSV on the chloroplast structure of rice leaves

**Figure 7:**
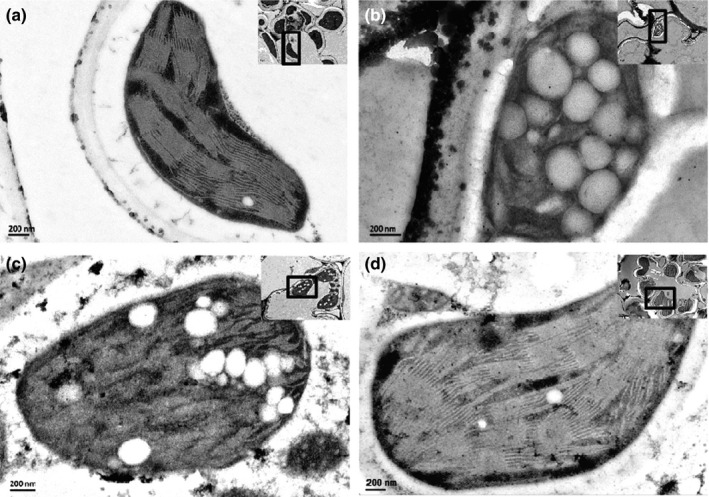
Effect of insect‐derived and plant‐derived Rice stripe virus (RSV) on the chloroplast of rice leaves (*Oryza sativa*). Colloidal gold immunoelectron micrographs of (a) healthy rice leaves, (b) rice leaves fed on by viruliferous small brown planthoppers (*Laodelphax striatellus*), (c) microinjected with RSV crude preparations from viruliferous small brown planthoppers, or (d) rice leaves from RSV‐infected rice seedlings. Anti‐Cp monoclonal antibody and 10 nm gold‐conjugated goat‐anti‐mouse IgG were used. The enlarged views of RSV particles are shown in Supporting Information Fig. S3.

**Figure 8:**
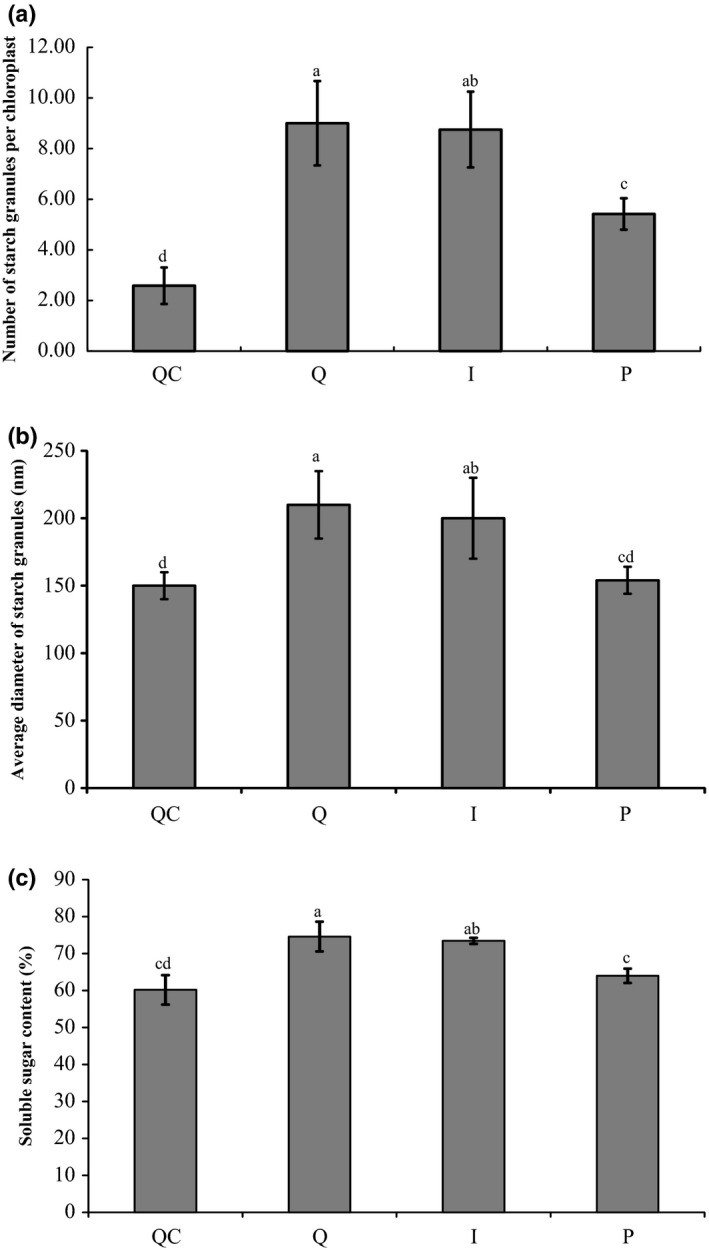
Effect of insect‐derived and plant‐derived Rice stripe virus (RSV) on (a) the number of starch granules, (b) average diameter of starch granules and (c) free sugar concentration of rice leaves (*Oryza sativa*). Q, rice leaves fed on by viruliferous planthoppers (*Laodelphax striatellus*); QC, rice leaves without planthopper infestation; I, rice leaves microinjected with insect‐derived RSV; P, rice leaves microinjected with plant‐derived RSV. The score was reported as mean ± SE. Differences were statistically evaluated using one‐way ANOVA followed by a Tukey's test. Different letters indicated significant differences at *P *<* *0.05 level.

In order to determine the effects of insect‐ and plant‐derived RSV on the chloroplast structure of rice leaves, the virus location in rice leaves was observed using colloidal gold immunoelectron microscopy after the leaves were infected by insect‐ or plant‐derived RSV. RSV of both origins invaded the chloroplasts (Fig. S3), but exhibited different effects on maintaining chloroplast structure. In cells of healthy rice and rice inoculated with plant‐derived RSV, the grana stacks are cystic structures which usually contain 10 to dozens of parallel sheets (Fig. 7a,d). However, the grana stacks in the chloroplast were apparently distorted in the leaves infected by viruliferous planthoppers or mechanically inoculated insect‐derived RSV. Only two to six parallel sheets were contained in each grana stack, and the whole grana stacks turned saucer‐shaped (Fig. 7b,c). The numbers of starch granules in the intact leaves, leaves infected by viruliferous planthoppers, and leaves mechanically inoculated with insect‐derived RSV or with plant‐derived RSV were 2.6 ± 0.7, 9.0 ± 1.7, 8.8 ± 1.5 and 5.4 ± 0.6, respectively (Fig. 8a). The average diameters of starch granules were 150 ± 10 (healthy leaves), 210 ± 25 (leaves infected by viruliferous planthoppers), 200 ± 30 (leaves mechanically inoculated with insect‐derived RSV) and 155 ± 10 nm (leaves mechanically inoculated with plant‐derived RSV) (Fig. 8b). Moreover, free sugar concentrations in rice leaves varied in the different treatments. The total contents of soluble sugar in the intact leaves, leaves infected by viruliferous planthoppers, leaves mechanically inoculated with insect‐derived RSV and with plant‐derived RSV were 60.2 ± 2.0%, 74.6 ± 2.0%, 73.4 ± 0.9% and 64.0 ± 1.4%, respectively (Fig. 8c). These results indicate that both insect‐ and, to a significantly lesser degree, plant‐derived RSV can enhance the number of starch granules and the content of soluble sugar. However, only insect‐derived RSV can destroy chloroplast structure and enlarge the starch granules.

## Discussion

Transmission between plant hosts through vectors is an important step in the biological cycle of plant viruses to ensure their maintenance and survival. As a persistent‐propagative virus, Rice stripe virus (RSV) has evolved to erect intimate and complicated interactions with its insect vectors. In this study, we disclosed the different disease symptoms and molecular responses in rice infected by insect‐ and plant‐derived RSV. Unlike insect‐derived RSV, plant‐derived RSV cannot produce typical stripe symptoms because of its incapacity to inhibit the expression of photosynthesis‐related genes and to sabotage chloroplast structure. The pathogenicity was acquired in both the alimentary canal and salivary gland of insect vectors, but the former showed stronger response, including immune response, to RSV than the latter.

We succeeded in mechanically inoculating RSV into rice leaves. Most persistent‐propagative viruses cannot be transmitted between plants through seeds or through wounds or abrasions in the plant surface. However, many persistent viruses in maize, such as maize rayado fino marafivirus and maize white line mosaic virus, have been successfully transferred to healthy plants through vascular puncture inoculation (Louie, 1995; Madriz‐Ordenana *et al*., 2000). Mechanical transmission of RSV usually fails (Ling, 1972) or yields a low infectious rate; in particular, only two out of 31 tested plants (*c*. 6%) are infected after injection with the sap of diseased leaves into the midrib of rice leaves with 0.01 M cysteine‐HCl solution (Okuyama & Asuyama, 1959). In the present study, the incidence of RSV by mechanical transmission increased to *c*. 17% after the midrib microinjection of insect‐derived RSV crude extracts. This rate is still much lower than that (*c*. 53%) of insect vector transmission. Nevertheless, our method provides a convenient means to fulfill a uniform RSV inoculum, which cannot be secured by insect vector transmission.

Plant‐ and insect‐derived RSV induce different disease symptoms in rice plants. Although the symptoms of RSV‐infected rice leaves vary with growth stages (Zhang *et al*., 2012), typical symptoms of RSV are discontinuous yellow stripes and dead tissue streaks on the leaves. Severe infections cause gray necrotic streaks and result in plant death. In contrast to insect‐derived RSV, plant‐derived RSV did not induce typical stripe symptoms but rather produced chlorosis or wilt in the whole local and systemic leaves. Chlorosis can be caused by different factors, including virus infection, nutrient deficiency, pest insect infection and planting problems. Virus‐infected leaves usually present patterned chlorosis, such as mosaic, mottle, streak, ringspot and line pattern; the underlying mechanism is the reduced photosynthetic efficiency caused by chloroplast function disruption (Cheng *et al*., 2008; Xu & Nagy, 2010). Our results demonstrated that plant‐derived RSV neither destroys chloroplast structure nor influences the transcription levels of photosynthesis‐related genes. Therefore, plant‐derived and insect‐derived RSV has different mechanisms of inducing plant chlorosis and different pathogenicities in plants.

Plant‐derived RSV is less likely to be transmitted to new hosts than insect‐derived RSV. For the seven genes of RSV, the relative RNA levels of *cp* and *ns3* were higher from plant‐derived RSV compared with insect‐derived RSV during rice infection. NS3 is a suppressor of gene silencing, thus enabling viruses escape the RNA silencing of the natural antiviral mechanism in plants (Soosaar *et al*., 2005; Xiong *et al*., 2008). The increase of *ns3* RNA level in the leaves inoculated with plant‐derived RSV facilitates RSV propagation that entails a high expression of *cp*. Such excessive propagation of RSV may consume too much nutrition of host plants, which leads to serious chlorosis and increases the risk of plant death. The reduction of rice lifespan would almost certainly result in a lack of further transmission. So we believe that plant‐derived RSV is not suitable for maintaining the dynamic balance in the insect–virus–plant system. Moreover, in contrast to plant‐derived RSV, insect‐derived RSV can greatly increase the total soluble sugar content of plant cells through expanded starch granules. The increased soluble sugar content may provide additional nutrients to insect vectors. Therefore, RSV takes advantage of insect vectors to acquire pathogenicity and broad spreading, and further modifies host plants to become more nutritious for insects. This reciprocity is probably an important factor for RSV in adopting the insect vector‐dependent transmission mode.

The pathogenicity acquisition of RSV could be dependent on the successful interaction between RSV Sp and plant PsbP. Sp is the disease‐specific protein that is strongly correlated with rice stripe disease symptoms (Zhu *et al*., 1992). Sp accumulates in the chloroplast, cytoplasm and nucleus in RSV‐infected rice cells, and the accumulation level of this protein positively correlates with the severity of RSV disease symptom (Zhu *et al*., 1992). Recent studies have proven that Sp interacts with PsbP, a photosystem II subunit that maintains the integrity of grana stacks in the chloroplast (Yi *et al*., 2007; Roose *et al*., 2011; Kong *et al*., 2014). In the present study, plant‐derived RSV did not suppress PsbP, and further study is required to determine if its Sp can interact effectively with PsbP.

In conclusion, we disclosed the different pathogenicities of RSV from insect vectors and host plants. The different responses of the salivary gland and the alimentary canal to RSV and the different effects of insect‐ and plant‐derived RSV on plants provided insights into the pathogenic mechanisms of persistent phytoviruses and even insect‐borne human viruses.

## Author contributions

W.Z. performed all lab experiments. P.Y. did the transcriptome analysis. F.C. and L.K. designed the experiments. W.Z., F.C. and L.K. wrote the manuscript.

## Supporting information

Please note: Wiley Blackwell are not responsible for the content or functionality of any supporting information supplied by the authors. Any queries (other than missing material) should be directed to the *New Phytologist* Central Office.

**Fig. S1** Quantification of Rice stripe virus (RSV) proliferation in alimentary canals and salivary glands of small brown planthoppers.
**Fig. S2** Pathogenicity analysis of plant‐derived Rice stripe virus (RSV).
**Fig. S3** Localization of Rice stripe virus (RSV) particles in the chloroplasts of rice leaves.
**Table S1** Primers used in this study
**Table S2** Statistical data for RNA‐seq reads of *Oryza sativa* mapped to gene set
**Table S3** Downregulated photosynthesis‐related genes of *Oryza sativa* fed on by viruliferous small brown planthoppers or microinjected with Rice stripe virus (RSV) crude preparations from viruliferous planthoppers
