# Supplementary material for: Different pathogenicities of Rice stripe virus from the insect vector and from viruliferous plants
Source: New Phytol. 2015 Nov 20;210(1):196–207. doi: 10.1111/nph.13747 (PMC5063192; doi:10.1111/nph.13747)
Supplement: Supplementary file 1 — Fig. S1 Quantification of Rice stripe virus (RSV) proliferation in alimentary canals and salivary glands of small brown planthoppers. Fig. S2 Pathogenicity analysis of plant‐derived Rice stripe virus (RSV). Fig. S3 Localization of Rice stripe virus (RSV) particles in the chloroplasts of rice leaves. Table S1 Primers used in this study Table S2 Statistical data for RNA‐seq reads of Oryza sativa mapped to gene set Table S3 Downregulated photosynthesis‐related genes of Oryza sativa fed on by viruliferous small brown planthoppers or microinjected with Rice stripe virus (RSV) crude preparations from viruliferous planthoppers [file NPH-210-196-s001.pdf]

New Phytologist

# *New Phytologist* Supporting Information Figs S1–S3 and Tables S1–S3

Article title: Different pathogenicities of Rice stripe virus from the insect vector and from viruliferous plants

Authors: Wan Zhao, Pengcheng Yang, Le Kang and Feng Cui

Article acceptance date: 6 October 2015

The following Supporting Information is available for this article:

**Fig. S1** Quantification of Rice stripe virus (RSV) proliferation in alimentary canals and salivary glands of small brown planthoppers.

**Fig. S2** Pathogenicity analysis of plant-derived Rice stripe virus (RSV).

**Fig. S3** Localization of Rice stripe virus (RSV) particles in the chloroplasts of rice leaves.

**Table S1** Primers used in this study

**Table S2** Statistical data for RNA-seq reads of *Oryza sativa* mapped to gene set

**Table S3** Downregulated photosynthesis-related genes of *Oryza sativa* fed by viruliferous small brown planthoppers or microinjected with Rice stripe virus (RSV) crude preparations from viruliferous planthoppers

**Fig. S1** Quantification of Rice stripe virus (RSV) proliferation in alimentary canals and salivary glands of small brown planthoppers. (a) Minimum acquisition access period of RSV in the two tissues after the planthoppers were fed on artificial diet containing plant-derived RSV crude preparations. The RNA level of *cp* was examined using quantitative real-time (qRT)-PCR and normalized by the transcript level of planthopper translation elongation factor 2 (*ef2*). The RNA level of *cp* was reported as mean ± SE. Differences were statistically evaluated using SPSS 17.0 by one-way ANOVA followed by a Tukey's test for multiple comparisons. (b) Proliferation of RSV up to 168 h in the two tissues examined by qRT-PCR based on the RNA level of *cp*. The transcript level of planthopper *ef*2 was used as an internal control. The RNA level of *cp* was reported as mean ± SE. Differences were statistically evaluated by t-test using SPSS 17.0. *, $P < 0.05$; **, $P < 0.01$. (c) Western blot analysis of Cp in the two tissues as a function of time. Monoclonal anti-Cp antibody and monoclonal anti-beta-tubulin antibody were used as the primary antibodies. (d) Gray scanning analysis of (c). The relative gray score was reported as mean ± SE. Differences were statistically evaluated using SPSS 17.0 by one-way ANOVA followed by a Tukey's test for multiple comparisons. Different letters indicated significant differences at $P < 0.05$ level.

# Fig. S1

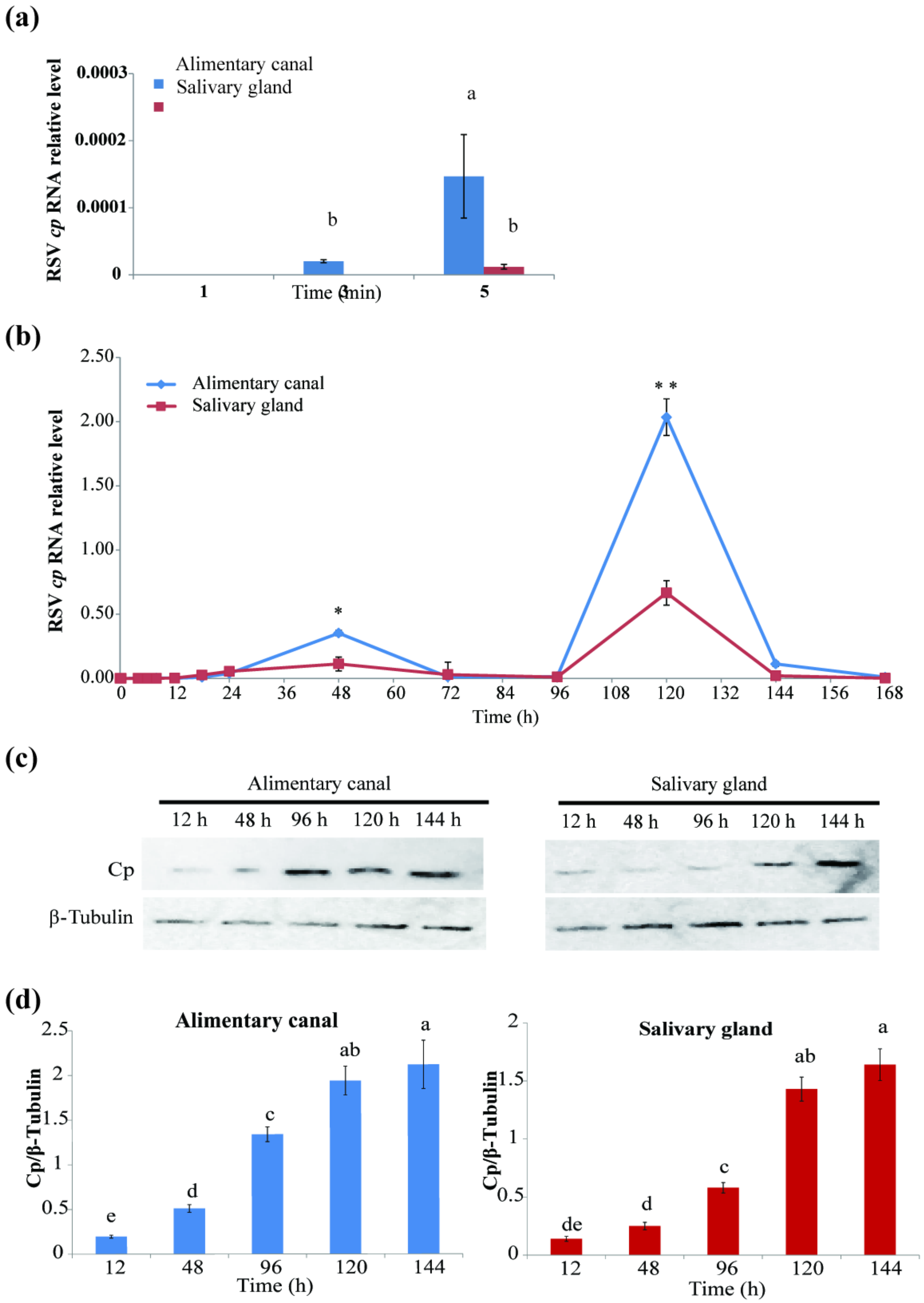
(a)
RSV cp RNA relative level
0.0003
0.0002
0.0001
0
Alimentary canal
Salivary gland
a
b
b
1
Time (min)
3
5
(b)
RSV cp RNA relative level
2.50
2.00
1.50
1.00
0.50
0.00
Alimentary canal
Salivary gland
* *
*
0
12
24
36
48
60
72
84
96
108
120
132
144
156
168
Time (h)
(c)
Alimentary canal
Salivary gland
12 h
48 h
96 h
120 h
144 h
Cp
β-Tubulin
(d)
Alimentary canal
Cp/β-Tubulin
2.5
2
1.5
1
0.5
0
e
d
c
ab
a
12
48
96
120
144
Time (h)
Salivary gland
Cp/β-Tubulin
2
1.5
1
0.5
0
de
d
c
ab
a
12
48
96
120
144
Time (h)

**Fig. S2** Pathogenicity analysis of plant-derived Rice stripe virus (RSV). (a) Western analysis of RSV in the virus crude preparations from viruliferous rice seedlings (P), viruliferous planthoppers (I), or the alimentary canal (AC) and salivary gland (SG) of viruliferous planthoppers. Monoclonal anti-Cp antibody was used as the primary antibody. Crude extracts from nonviruliferous planthoppers (IC) or nonviruliferous rice leaves (PC) served as negative controls. Beta-actin of rice leaves were used as internal controls. (b) Western blot analysis detection of RSV Cp in the injected and systemic leaves of rice that were microinjected with RSV crude preparations from viruliferous planthoppers (In-RSV) or viruliferous rice seedlings (P-RSV) after 6-wk or 8-wk culture, respectively, using monoclonal anti-Cp antibody. Beta-actin of rice was used as an internal control by immunoblotting against plant β-actin monoclonal antibody. (c) Disease symptom of rice leaves microinjected with RSV crude preparations from viruliferous rice seedlings with five dilution proportions (from 1 : 1 to 1 : 10000). (d) Disease symptom of rice leaves inoculated by the planthoppers that were fed on the artificial diet with RSV crude preparations from viruliferous rice or viruliferous planthoppers. (e) Disease symptom of rice leaves that were microinjected with plant-derived RSV and 2 d later fed by nonviruliferous planthoppers. The rice leaves microinjected with plant-derived RSV without planthopper infestation were used as negative control.

New Phytologist

# Fig. S2

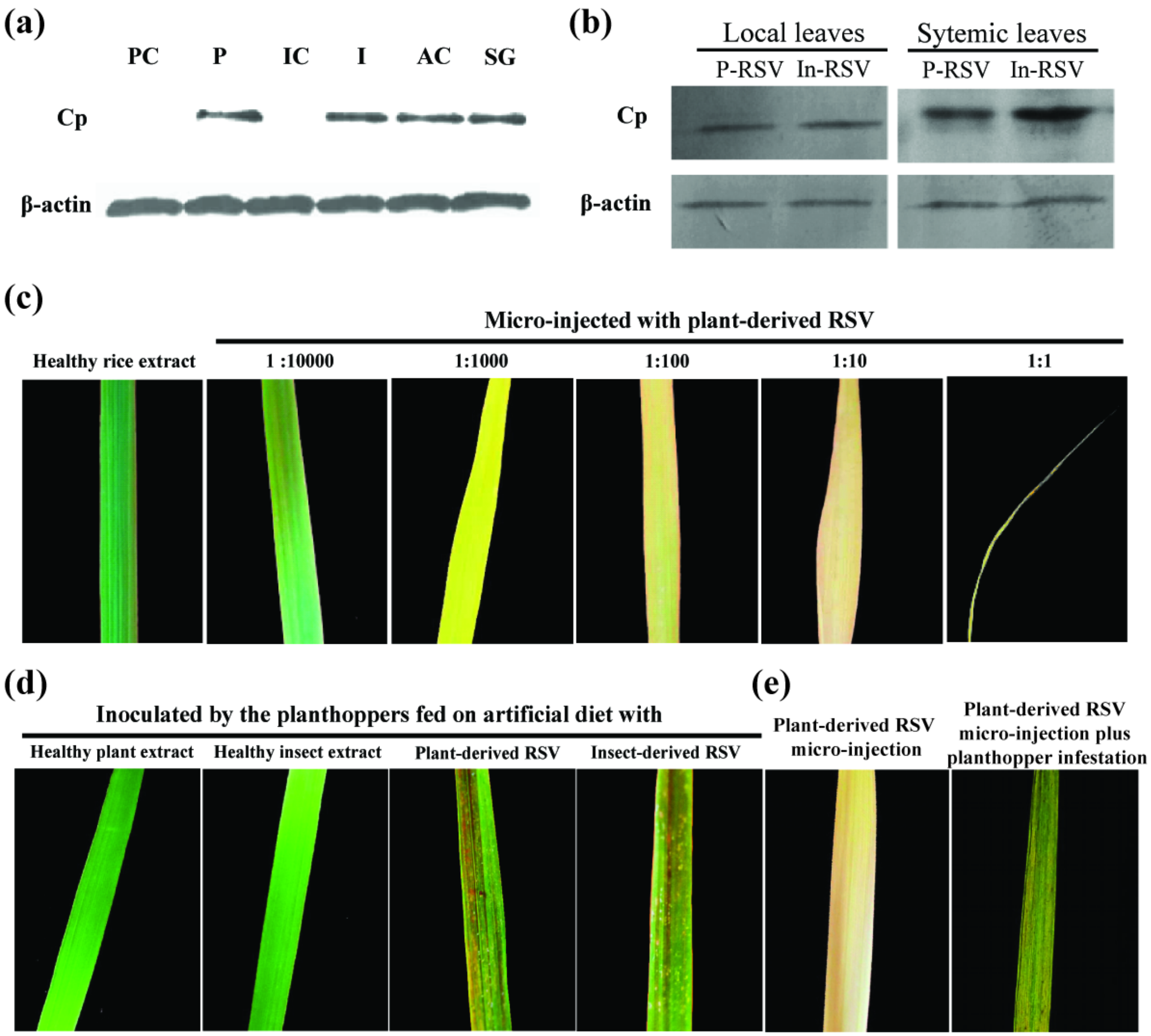

**Fig. S3** Localization of Rice stripe virus (RSV) particles in the chloroplasts of rice leaves. Colloidal gold immunoelectron micrographs of (a) healthy rice leaves, (b) rice leaves fed by viruliferous small brown planthoppers, (c) microinjected with RSV crude preparations from viruliferous planthoppers, or (d) from viruliferous rice seedlings. Anti-Cp monoclonal antibody and 10-nm gold-conjugated goat-anti-mouse IgG were used. Black triangles demonstrate the localization of RSV particles. Bars, 200 nm.

## Fig. S3

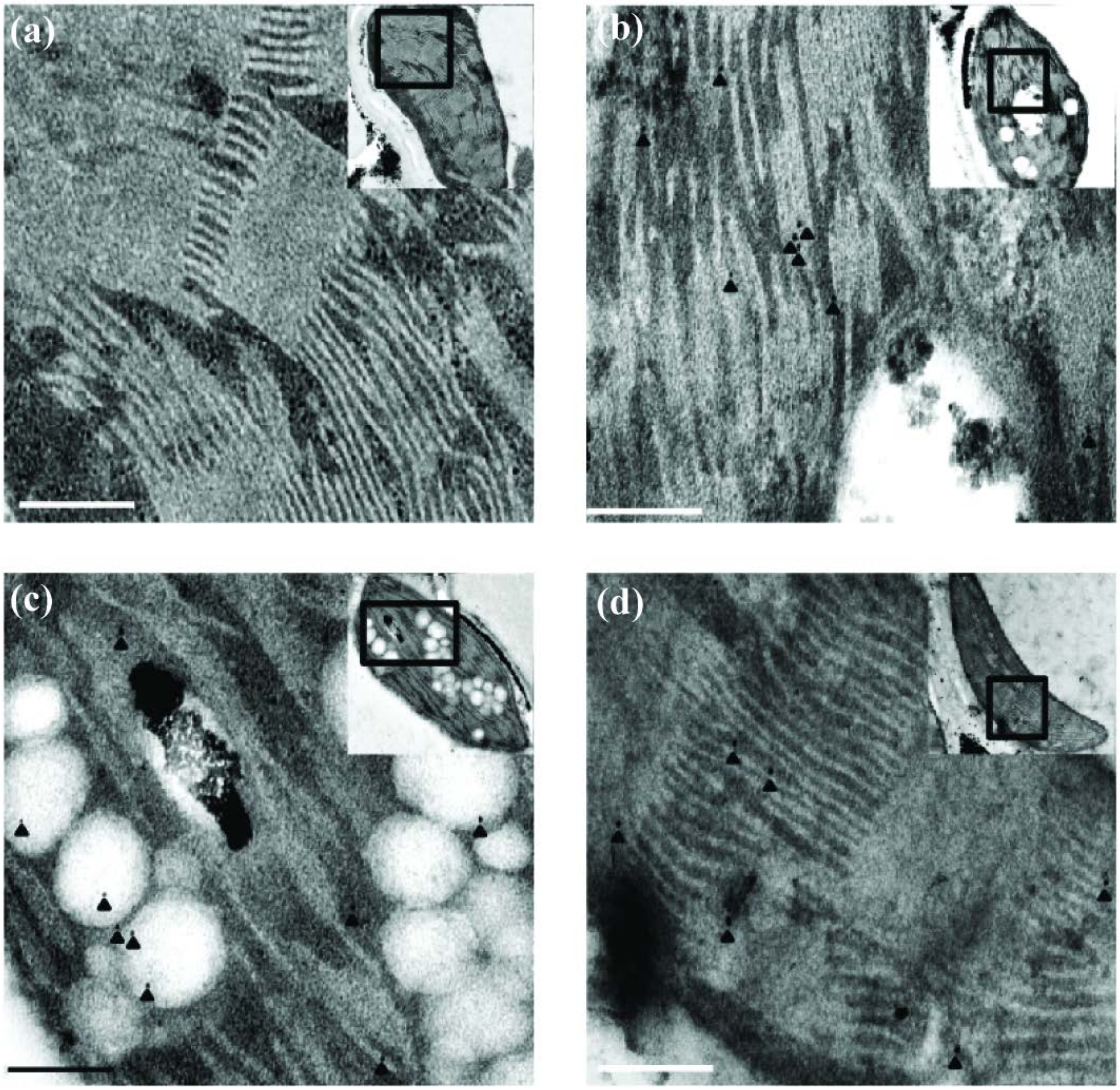

**Table S1** Primers used in this study

| | **Forward primer (F)** | **Reverse primer (R)** |
|---|---|---|
| ***cp*** | GGAATTACATATGATGGGTACC AACAAGCCAGC | CCGCTCGAGGTCATCTGCACCTTC TGCC |
| ***cp*-q** | AGTGCTGATCGTATTGACAGA | GATGAAGTACACAACTGGTC |
| ***sp*-q** | TGCAAGACGTACAAAGGACAA | TCTTCCGTGACTCTCTCTGG |
| ***nsvc2*-q** | CTTTCCCTGACACCCATTC | TCATAAGTCAATTTGCAGGG |
| ***ns2*-q** | TCTCTTCAATTTGAGGTGCTC | ATTCAGTTGCTATGGCGAG |
| ***ns3*-q** | TGACATCCATTCATCTAGGC | GGGACAGAATCCAGATCAGAG |
| ***nsvc4*-q** | GGAACCCTTCAGCTTCTCAG | TGGGTGAGAGGTTGATGAAA |
| ***rdrp*-q** | TTGGTACATACAACCCGGAA | ATCTCCTGACCTCCATCTGC |
| ***ef2*-q** | GTCTCCACGGATGGGCTTT | ATCTTGAATTTCTCGGCATACATTT |
| ***ubq*-q** | TCACCTACGTCTACAACCAG | AGTGCTGATCGTATTGACAGA |
| ***psbp*-q** | GGGAAGCCCAAGACGAACAC | CTCGGTGATGGTCTTCTTGG |

**Table S2** Statistical data for RNA-seq reads of *Oryza sativa* mapped to gene set

| Sample | GC% | Q30% | Clean reads | Total mapped | %Total mapped | Unique mapped | %Unique mapped |
|---|---|---|---|---|---|---|---|
| **I1** | 50 | 97.42 | 12,426,308 | 11,406,238 | 91.7 | 9,600,968 | 77.2 |
| **I2** | 52 | 97.11 | 17,895,791 | 16,499,006 | 92.1 | 13,828,393 | 77.2 |
| **I3** | 52 | 97.49 | 11,919,666 | 10,959,492 | 91.9 | 9,323,743 | 78.2 |
| **IC1** | 52 | 97.29 | 11,164,750 | 10,057,000 | 90.0 | 8,314,319 | 74.4 |
| **IC2** | 51 | 97.51 | 11,915,974 | 10,845,925 | 90.9 | 8,239,885 | 69.1 |
| **IC3** | 52 | 97.28 | 14,117,504 | 12,862,088 | 91.0 | 9,888,800 | 70.0 |
| **QC1** | 52 | 97.30 | 11,595,329 | 10,507,480 | 90.5 | 8,431,750 | 72.7 |
| **QC2** | 51 | 97.27 | 11,418,861 | 10,242,061 | 89.6 | 8,275,365 | 72.4 |
| **QC3** | 51 | 98.19 | 14,693,794 | 13,236,848 | 90.0 | 11,625,795 | 79.0 |
| **P1** | 52 | 97.23 | 15,490,017 | 14,171,010 | 91.4 | 11,532,767 | 74.4 |
| **P2** | 52 | 97.22 | 14,276,877 | 13,032,776 | 91.2 | 10,927,179 | 76.5 |
| **P3** | 52 | 97.26 | 14,716,545 | 13,563,757 | 92.1 | 10,989,574 | 74.6 |
| **PC1** | 52 | 97.24 | 10,480,638 | 9,507,307 | 90.6 | 7,517,223 | 71.7 |
| **PC2** | 51 | 97.32 | 10,687,140 | 9,486,918 | 88.7 | 7,841,224 | 73.3 |
| **PC3** | 51 | 97.47 | 11,185,037 | 10,113,001 | 90.3 | 7,932,192 | 70.9 |
| **Q1** | 51 | 98.27 | 12,891,834 | 11,481,097 | 89.0 | 10,181,685 | 78.9 |
| **Q2** | 52 | 98.05 | 12,205,722 | 10,952,580 | 89.7 | 9,650,889 | 79.0 |
| **Q3** | 51 | 97.61 | 12,742,079 | 11,619,695 | 91.1 | 10,403,220 | 81.6 |

Q, rice leaves fed by viruliferous planthoppers; QC, rice leaves without planthopper infestation; I, rice leaves microinjected with insect-derived RSV; IC, rice leaves microinjected with extracts of nonviruliferous planthoppers; P, rice leaves microinjected with plant-derived RSV; PC, rice leaves microinjected with extracts of healthy plants. Three biological replicates were sequenced for each group.

**Table S3** Downregulated photosynthesis-related genes of *Oryza sativa* fed by viruliferous small brown planthoppers or microinjected with Rice stripe virus (RSV) crude preparations from viruliferous planthoppers

| GeneID | LogFC (I/IC) | LogFC (Q/QC) | LogFC (P/PC) | Annotation |
|---|---|---|---|---|
| **Photosynthesis - antenna proteins** | | | | |
| **LOC_Os04g38410.1** | -1.09* | -1.94* | 0.18 | Light-harvesting complex II chlorophyll a/b binding protein proteins (LHCB6) |
| **LOC_Os11g1389.1** | -1.09* | -2.61* | -0.25 | Light-harvesting complex II chlorophyll a/b binding protein 5 (LHCB5) |
| **LOC_Os07g37240.1** | -1.07* | -2.66* | -0.03 | Light-harvesting complex II chlorophyll a/b binding protein 4 (LHCB4) |
| **LOC_Os07g37550.1** | -1.06* | -2.11* | 0.03 | Light-harvesting complex II chlorophyll a/b binding protein 3 (LHCB3) |
| **LOC_Os03g39610.1** | -1.05* | -2.51* | 0.15 | Light-harvesting complex II chlorophyll a/b binding protein 2 (LHCB2) |
| **LOC_Os01g52240.1** | -1.07* | -3.45* | 0.21 | Light-harvesting complex II chlorophyll a/b binding protein 1 (LHCB1) |
| **LOC_Os08g33820.1** | -1.02* | -2.19* | -0.30 | Light-harvesting complex I chlorophyll a/b binding protein 4 (LHCA4) |
| **LOC_Os02g10390.1** | -1.16* | -1.37* | -0.32 | Light-harvesting complex I chlorophyll a/b binding protein 3 (LHCA3) |
| **LOC_Os07g38960.1** | -1.02* | -1.84* | 0.25 | Light-harvesting complex I chlorophyll a/b binding protein 2 (LHCA2) |
| **LOC_Os06g21590.1** | -1.11* | -2.36* | 0.10 | Light-harvesting complex I chlorophyll a/b binding protein 1 (LHCA1) |
| **Photosynthesis** | | | | |
| **LOC_Os12g37710.1** | -1.34* | -1.13* | -0.27 | Photosystem II oxygen-evolving enhancer protein 2 (psbP) |
| **LOC_Os07g05360.1** | -2.15* | -6.28* | 0.80* | Photosystem II 10kDa protein (psbR) |
| **LOC_Os08g02630.1** | -1.07* | -2.00* | -0.36 | Photosystem II PsbY protein |
| **LOC_Os04g33830.1** | -1.00* | -1.98* | -0.18 | Photosystem I subunit PsaO |
| **LOC_Os12g08770.1** | -1.09* | -1.95* | 0.07 | Photosystem I subunit PsaN |
| **LOC_Os07g05480.1** | -1.00* | -1.74* | 0.04 | Photosystem I subunit X (psaG/psaK) |
| **LOC_Os01g31690.1** | -1.22* | -1.73* | 0.08 | Photosystem II oxygen-evolving enhancer protein 1 (psbO) |
| **LOC_Os08g01380.1** | -1.02* | -1.72* | -0.17 | Ferredoxin (petF) |
| **LOC_Os09g30340.1** | -1.07* | -1.59* | -0.08 | Photosystem I subunit V (psaG) |
| **LOC_Os10g38272.1** | -3.65* | -2.64* | 0.00 | F-type H+-transporting ATPase subunit b (atpF) |
| **Porphyrin and chlorophyll metabolism** | | | | |

| | | | | |
|---|---|---|---|---|
| **LOC_Os02g51080.1** | -1.86* | -0.87* | 0.00 | FAD binding domain containing protein, expressed |
| **LOC_Os03g36540.1** | -1.79* | -1.27* | 0.09 | magnesium-chelatase subunit chlI, chloroplast precursor, putative, expressed |
| **LOC_Os03g20700.1** | -1.73* | -0.71* | -0.24 | magnesium-chelatase, putative, expressed |
| **LOC_Os06g04150.1** | -1.94* | -0.60* | 0.10 | magnesium-protoporphyrin O-methyltransferase, putative, expressed |
| **LOC_Os10g35370.1** | -1.40* | -0.73* | 0.53 | oxidoreductase, short chain dehydrogenase/reductase family domain containing family, expressed |
| **LOC_Os04g58200.1** | -2.82* | -1.05* | 0.98* | protochlorophyllide reductase A, chloroplast precursor, putative, expressed |

*, FDR $<0.01$ FC, fold change; Q, rice leaves fed by viruliferous planthoppers; QC, rice leaves without planthopper infestation; I, rice leaves microinjected with insect-derived RSV; IC, rice leaves microinjected with extracts of nonviruliferous planthoppers; P, rice leaves microinjected with plant-derived RSV; PC, rice leaves microinjected with extracts of healthy plants.
